# *Lactobacillus rhamnosus* strains of oral and vaginal origin show strong antifungal activity *in vitro*

**DOI:** 10.1080/20002297.2020.1832832

**Published:** 2020-10-18

**Authors:** Mette Rose Jørgensen, Pernille Thestrup Rikvold, Mads Lichtenberg, Peter Østrup Jensen, Camilla Kragelund, Svante Twetman

**Affiliations:** a Department of Odontology, Faculty of Health and Medical Sciences, University of Copenhagen, Copenhagen, Denmark; b Costerton Biofilm Center, Department of Immunology and Microbiology, University of Copenhagen, Copenhagen, Denmark; c Department of Clinical Microbiology, Copenhagen University Hospital, Copenhagen, Denmark

**Keywords:** Probiotic bacteria, yeasts, candidosis, growth inhibition, pH, strain-specificity

## Abstract

**Background:** Intake of probiotic bacteria may prevent oral *Candida* infection.

**Objective:** To screen the antifungal activity of 14 *Lactobacillus* candidate strains of human origin, against six opportunistic *C. albicans* and non-albicans species. A second aim was to study the acid production of the four strains showing the strongest antifungal activity.

**Methods:** We used an agar overlay growth inhibition assay to the assess the antifungal activity of the lactobacilli. The acid-producing capacity was measured with pH micro-sensors.

**Results:** All 14 *Lactobacillus* candidates inhibited the growth of the *Candida* spp. The four best-performing strains were *L. rhamnosus* DSM 32992 (oral origin), *L. rhamnosus* DSM 32991 (oral), *L. jensenii* 22B42 (vaginal), and *L. rhamnosus* PB01 (vaginal). The difference between *L. rhamnosus* DSM 32992 and the other three strains was statistically significant (p < 0.001). The *Candida* spp. differed in susceptibility; *C. parapsilosis* was highly inhibited, while *C. krusei* was not or slightly inhibited. The oral *L. rhamnosus* DSM 32992 and DSM 32991 strains showed the lowest pH-values.

**Conclusion:** Screening of probiotic lactobacilli showed significant strain-dependent variations in their antifungal capacity in a pH-dependent mode. Two strains of oral origin were most effective. A further characterization seems justified to elaborate on their probiotic properties.

## Introduction

*Candida* species (spp.) are ubiquitous commensals taking part of a healthy oral microbiota. *C. albicans* is the predominant specie and is believed to be present in its natural blastospore form in around 50% of the healthy population [1,2]. In healthy individuals, the oral microbiota appears to prevent *Candida* spp. to shift from harmless commensals to disease-causing pathogens. However, certain conditions may disrupt the homeostatic bacteriome–mycobiome relationship and predispose the *Candida* spp. to become pathogenic and cause disease in the oral mucosa, termed oral candidosis [3]. While *C. albicans* is the main contributor to infection, the incidence of oral infection due to non-*albicans* spp. have increased over the last decades, and the most frequently isolated strains associated with disease are *C. glabrata, C. krusei, C. tropicalis, C. dubliniensis*, and *C. parapsilosis* [4,5].

Currently, treatment options of oral candidosis include topical and systemic antimycotics. However, the American Centers for Disease Control and Prevention has reported that about 7% of all *Candida* bloodstream isolates are resistant to fluconazole [6]. Although *C. albicans* is the most common cause of severe *Candida* infections, resistance is most common in other species, particularly *C. glabrata* and *C. parapsilosis* [6]. According to the World Health Organization, *Candida* infection is a significant concern for human health in vulnerable populations due to the emergence of new resistance mechanisms in the microorganisms. In most countries where data are available, drug resistance appears to be higher among non-*albicans* species than among *C. albicans* [7]. Hence, the search of alternative antimycotic treatments to prevent and combat oral mucosal infections of both *C. albicans* and non-*albicans* spp. is highly relevant.

Probiotic bacteria have been proposed as a supplement to conventional therapies to prevent and combat oral *Candida*-infection [8–10]. Probiotic bacteria are defined as ‘live microorganism that, when administered in adequate amounts, confer a health benefit on the host’ [11]. The most investigated probiotics for anti-*Candida* activity belong to the genera *Lactobacillus* and *Bifidobacterium*, but also *Streptococcus* has been proposed [12]. Laboratory studies have shown that probiotic lactobacilli can inhibit *C. albicans* growth [13,14], interfere with the *C. albicans* biofilm formation on dentures [15], and to interfere in hyphae formation [16]. In addition, Tan and coworkers [17] showed that sterile spent media of *L. gasseri* BF and *L. rhamnosus* BF could disrupt biofilm formation of *C. tropicalis* BF, *C. krusei* BF, and *C. parapsilosis* BF and reduce their metabolic activity. It has also been demonstrated that cell-free supernatants of the probiotic *L. pentosus* strain LAP1 could inhibit the growth of *C. albicans, C. tropicalis*, and *C. krusei in vitro* [18].

Several clinical trials have confirmed that regular probiotic administration can decrease the *Candida* cell counts in samples from the oral cavity, in particular among elderly individuals prone to infections [8,19–22] and be a possible supplement to conventional antimycotic treatment [23,24]. However, the genera of probiotics used in the trials are wide-spread, and the choice of an appropriate type of probiotic for this specific target is challenging. Recently, we showed significant differences in the antifungal properties of two *L. reuteri* strains (ATCC PTA 5289 and DSM 17938) [9]. This finding emphasizes that the probiotic strain-specificity cannot be disregarded, and that the search for appropriate probiotic strains with strong anti-*Candida* properties for oral application is warranted. The primary aim of this study was, therefore, to screen the antifungal activity of 14 selected probiotic *Lactobacillus* candidate strains, isolated from the human oral cavity or vagina, against six opportunistic *Candida* spp. *in vitro*. A secondary aim was to study the acid production abilities of the four best-performing lactobacilli in the growth inhibition assay with aid of pH microsensor measurements through *Candida* and *Lactobacillus* co-cultures.

## Materials and methods

### Strains and culture conditions

Fourteen selected *Lactobacillus* spp. (Deerland Probiotics & Enzymes A/S, Hundested, Denmark) were used in this study together with six clinical *Candida* spp. from the Department of Clinical Microbiology, Copenhagen University Hospital, Copenhagen, Denmark, and six control spp. from the Culture Collection, University of Gothenburg, Sweden (Table 1). The lactobacilli were initially cultured on de Man Rogosa Sharpe (MRS) agar (Oxoid Ltd., Basingstoke, Hampshire, UK) for 24 hours (h) in an anaerobic chamber at 37°C (10% H_2_, 5% CO_2_ and 85% N_2_). The *Candida* strains were cultured on BD Difco™ Sabouraud Dextrose (SD) agar (Becton, Dickinson and Company, Sparks, MD, USA) for 24 h in ambient air at 37°C.

**Table 1.** List of *Lactobacillus* spp. and *Candida* spp. used in the study

Genus	Origin	Species
*Lactobacillus*	Oral cavity Vaginal	*L. rhamnosus* NEU427 *L. rhamnosus* ERB18, DSM 32,991 *L. rhamnosus* ERB 36, DSM 32,992 *L. fermentum* S1P2 *L. fermentum* S1P1 *L. curvatus* EB10 DSM 32,307 *L. paracasei* S1-P3 *L. acidophilus* EB03 *L. crispatus* 23B33 *L. crispatus* NEU458 DSM15224 *L. gasseri* EB01 *L. jensenii* 12B1 *L. jensenii* 22B42 *L. rhamnosus* PB01
*Candida*	Control CCUG strains Clinical isolates	*C. albicans* CCUG 46,390 *C. dubliniensis* CCUG 48,722 *C. glabrata* CCUG 63,819 *C. krusei* CCUG 56,126 *C. parapsilosis* CCUG 56,136 *C. tropicalis* CCUG 47,037 *C. albicans* CBS 562 NT *C. dubliniensis* 41_3 ZZMK *C. glabrata* CBS 863 *C. krusei* RV 491 *C. parapsilosis* 26 PBS *C. tropicalis* DSM 7524

### Growth inhibition assay

The growth inhibition assay was performed as described earlier [9]. In brief, one distinct colony of overnight cultured lactobacilli was transferred to 5 mL MRS broth (Oxoid Ltd., Basingstoke, Hampshire, UK) and incubated at 37°C for 24 h under anaerobic conditions. The following day the lactobacilli were harvested by centrifugation at 2,000 rpm for 10 min at room temperature. The supernatants of the *Lactobacillus* strains were obtained after centrifugation and destroyed. The pellets were washed three times in phosphate-buffered saline (PBS), and the OD was adjusted to 1.8 at 630 nm (Genesys™ 10S UV-Vis Spectrophotometer, Thermo Fisher Scientific, Waltham, MA, USA), corresponding to approximately 10^9^ cfu/mL. The cultures were then serially diluted in MRS broth in sixfold steps. One mL of the supernatants, undiluted suspensions and cell suspensions corresponding to approximately 10^7^ and 10^5^ CFU/mL were added to 24 mL sterilized molten MRS agar (~45°C) in Petri dishes and the agar could solidify. The plates were incubated overnight at 37°C under anaerobic conditions. One single colony of each of the overnight cultured *Candida* strains was added to 5 mL broth and aerobically incubated at 37°C for 24 h. The following day, one additional layer of 25 mL of molten sterile SD agar was poured on top of the MRS agar with grown lactobacilli and could solidify and air-dry for 3 h in room temperature. The overnight cultured *Candida* strains were diluted in SD broth to a final OD of 0.2 at 500 nm. The *Candida* suspensions were stamped on the plates with a Steers steel-pin replicator (CMI-Promex ICN, Pedricktown, NJ, USA) and left to dry for 2 h at room temperature. The plates were subsequently aerobically incubated overnight at 37°C. As controls, the *Candida* strains were stamped on the top of plates with no lactobacilli within the bottom MRS agar layer.

The assays were carried out in duplicates and repeated three times on different occasions. The results were evaluated with a 4-step score, modified from Simark-Mattson et al. [25]: Score 0 = complete growth inhibition (no visible colonies), Score 1 = almost total growth inhibition (colonies slightly visible), Score 2 = slight growth inhibition (colonies are clearly visible but smaller than at the control plate), and Score 3 = no growth inhibition (colonies equal to those at the control plate). Two observers (MRJ and PTR) scored the plates independently, and in case of disagreement, consensus was reached through discussion.

### Microsensor measurement of pH

Acid production (measured by pH) of the four best-performing lactobacilli in the growth inhibition assay and the effect of pH on *Candida* growth was measured with pH microelectrodes (pH-100 and ref-100; Unisense A/S, Århus, Denmark) with a linear response between pH 4–9 and a 90% response time of <10 s and a detection limit of 0.01 pH units. Measurements were performed using a modification of a previously described procedure [9] in selected plates from the growth inhibition test. In brief, the electrodes were mounted in a motorized PC-controlled profiling setup (MM33 and MC-232, Unisense A/S), and positioning and data acquisition were controlled by dedicated software (SensorTrace Profiling, Unisense A/S). The pH microsensor was calibrated against buffers of pH 4 and 7 at room temperature with a linear slope of −56.1 mV/pH unit. The pH was measured on the final day of the growth inhibition test. Before measuring, the pH and reference electrodes were placed approximately 2 mm above the surface of the *Candida* colonies. The pH was measured from the surface of the colonies in 100 µm increments until the depth of maximum 4.5 mm into the agar to make sure the sensors had reached the bottom agar layer containing the lactobacilli. Selection of the plates was based on the results from the interference test; the plates with the highest inhibition scores for each *Lactobacillus* spp. were selected regardless of concentration (CK and PØJ). The pH was measured through dense, slightly inhibited colonies of *Candida* (*C. krusei* and *C. tropicalis*) and through vague, almost completely inhibited colonies (*C. albicans, C. glabrata, C. dubliniensis*, and *C. parapsilosis*) incubated on plates with *L. rhamnosus* DSM 32992, *L. rhamnosus* DSM 32991, *L. jensenii* 22B42, and *L. rhamnosus* PB01, respectively. In addition, pH was measured in control plates containing only the lactobacilli in the bottom agar layer, in a control plate with only *Candida* strains, and lastly in a control agar plate without lactobacilli or *Candida* strains.

### Statistical analyses

All data were processed with SAS Enterprise guide software (version 7.1, SAS Institute Inc., Cary, NC, USA). A p-value <0.05 (two-sided) was considered statistically significant. For descriptive statistics, the growth inhibition scores for the lactobacilli at different doses are presented as the median score. The frequency of growth inhibition scores 0–3 for each *Lactobacillus* strain, for each dose, and for each *Candida* strain is presented in percentage distribution. A Poisson regression model was employed to test the influence of the *Lactobacillus* strains, *Candida* strains and dose variables on the growth inhibition score. Based on the results from the model, the four lactobacilli displaying the lowest growth inhibition scores were subjected to Chi-square tests.

## Results

### Growth inhibition

**Figure 1.**
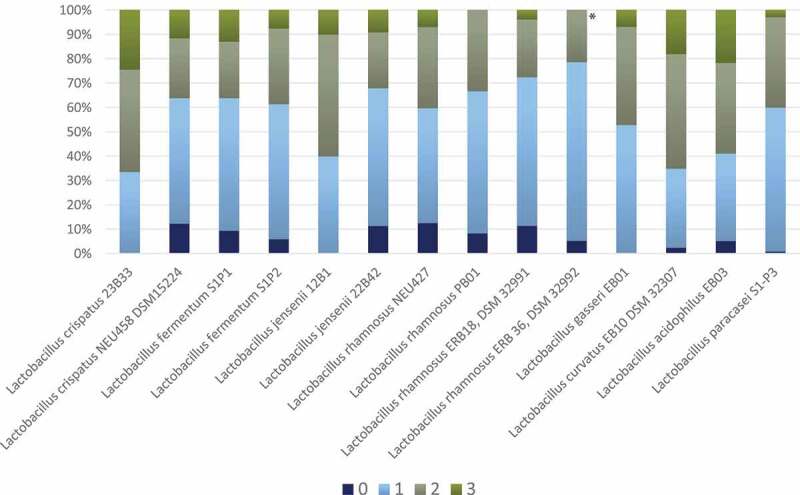
Frequency distribution (%) of growth inhibition score 0–3 for each *Lactobacillus* spp. (all concentrations and all *Candida* spp.). Results are based on duplicate assays, repeated at three separate occasions

**Figure 2.**
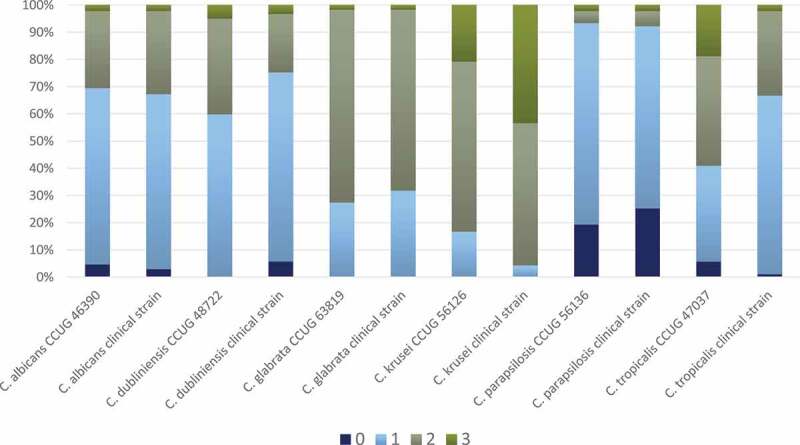
Frequency distribution (%) of growth inhibition score 0–3 for each *Candida* spp. based on agar overlay interference tests with all 14 *Lactobacillus* spp. (all concentrations). Results are based on duplicate assays, repeated at three separate occasions

All the 14 *Lactobacillus* strains proved to inhibit the growth of the *Candida* spp., but some were more effective than others, with a substantial *Candida* ssp. inhibition variation (Figure 1, Suppl. Table S1). The four best-performing lactobacilli were *L. rhamnosus* DSM 32992, which demonstrated the highest number of score 0 and 1, followed by *L. rhamnosus* DSM 32991, *L. jensenii* 22B42, and *L. rhamnosus* PB01. The difference between *L. rhamnosus* DSM 32992 and the other three strains was statistically significant (p < 0.001). In general, the growth inhibition was almost equal for the cell concentrations 10^9^ and 10^7^ CFU/mL, while the lowest concentration (10^5^ CFU/mL) appeared to be least effective (Suppl. Table S1, Suppl. Figure S1). The various *Candida* spp. displayed contrasting susceptibility; the growth of the clinical and control strains of *C. parapsilosis* was highly inhibited, while the two *C. krusei* strains presented no growth inhibition or only slight growth inhibition (Figure 2, Suppl. Table S1). The clinical *Candida* isolates and the control strains of the same species presented a similar susceptibility in the assays except for *C. tropicalis* for which the clinical isolate seemed to be more inhibited than the control strain.

*L. rhamnosus ERB 36 DSM 32992 demonstrated the best growth inhibition properties against the *Candida* spp. displaying the highest number of score 0 and 1 (p < 0.001), followed by *L. rhamnosus* DSM 32991, *L. jensenii* DSM 32558, and *L. rhamnosus* PB01, respectively

There was a statistically significant difference in growth susceptibility between the 12 *Candida* strains (p < 0.001). *C. parapsilosis* CCUG 56136 and *C. parapsilosis* clinical strain were growth inhibited to the highest extent displaying the highest number of score 0 and 1, while *C. krusei* CCUG 47037 and *C. krusei* clinical strain were only slightly inhibited or not inhibited displaying the highest number of score 2 and 3

### Microsensor pH measurements

**Figure 3.**
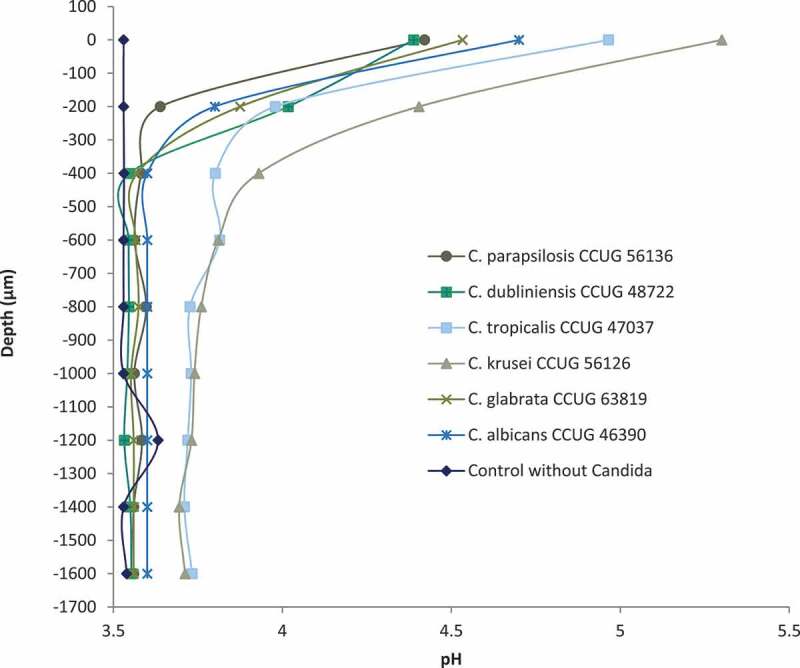
Micro-sensor measurement of pH with selected *Candida* strains with *L. rhamnosus* DSM 32992 (10^7^ CFU/mL) in the bottom agar layer of the co-cultured agar overlay assay. Zero on the vertical axis represents the first measurement from the sensor of either a *Candid*a colony or agar. No changes in pH values appeared for any of the strains at deeper levels in the agar

Microsensor measurements of pH were performed for the four above-mentioned best-performing *Lactobacillus* strains: *L. rhamnosus* DSM 32992, *L. rhamnosus* DSM 32991, *L. jensenii* 22B42, and *L. rhamnosus* PB01, of which the two former were isolated from the oral cavity and the two latter had vaginal origin. The pH of the plain double-layered agar was 5.8, and pH measurements from *L. rhamnosus* DSM 32992, *L. rhamnosus* DSM 32991, and *L. rhamnosus* PB01 generally showed lower pH values than *L. jensenii* 22B42 ranging from 3.5 to 3.8 (Suppl. Figure S2). In plates with only *Candida* spp. incubated, pH measured through dense colonies of *C. krusei* and *C. tropicalis* was approximately 6.8 at the surface of the colonies, reaching 5.8 around 700–1,000 µm down in the agar and remained stable. A similar picture was obtained when the pH-value was measured through *C. albicans* colonies, but the pH drop seemed to be more rapid, reaching 5.8 approximately 500 µm inside the agar. The measurements for *L. rhamnosus* DSM 32992 (10^7^ CFU/mL) through *C. albicans* and non-*C. albicans* colonies are presented in Figure 3. The surface pH was substantially lower compared with the *Candida* spp. incubated plates and the pH dropped rapidly through the first 400 µm. However, for the *C. krusei* and *C. tropicalis* reference strains, this drop was delayed and less dramatic (Figure 3). The pH profiles of the remaining three *Lactobacillus* strains inoculated with *Candida* spp. at the top agar layer showed very similar patterns (data not shown).

## Discussion

During recent years, the use of probiotic bacteria to control oral *Candida* colonization has been investigated as an adjunct therapeutic strategy to prevent infections in susceptible individuals [24,26,27]. In this study, we investigated the growth inhibition of 14 selected *Lactobacillus* strains isolated from the human oral cavity or vagina. We found a clear but strain-dependent effect on *Candida* growth which was in harmony with several previous *in vitro* experiments [9,17,26,28,29]. Interestingly, three of the best performing strains were *L. rhamnosus* strains, and the two best growth inhibitors, *L. rhamnosus* DSM 32992 and *L. rhamnosus* DSM 32991, were isolated from the oral cavity. *L. rhamnosus* is one of the most investigated probiotic *Lactobacillus* spp. and especially, the strain *L. rhamnosus* GG ATCC 53103 (LGG), isolated from a healthy human gastrointestinal tract, has shown remarkable probiotic properties [30,31]. Oral candidosis is a result of bacterial-fungal dysbiosis appearing when the microbial homeostasis is disrupted due to antibiotic treatment, local or systemic immune disturbances, smoking, hyposalivation, use of dentures, and poor oral hygiene [32]. To our knowledge, only one randomized clinical trial (RCT) has investigated the antifungal effect of a single strain of *L. rhamnosus* in the oral cavity [22]. When added to milk, *L. rhamnosus* SP1 provided a significant reduction in the severity of denture stomatitis and significantly reduced *Candida* counts in the probiotic group after 6 months’ intervention [22]. Four RCT’s [19–21,33] have shown decreased *Candida* counts after intervention with different *L. rhamnosus* strains; however, in these trials the strains were combined with non-*L. rhamnosus* probiotic species, and the antifungal effect can therefore not completely be ascribed to one probiotic strain. The beneficial clinical effects are however supported by laboratory studies showing that *L. rhamnosus* strains can protect oral epithelial tissue from damage, caused by *C. albicans*, by impacting its major virulence factors, including adhesion, competition for nutrients, invasion and hyphae formation [26,30,34].

The probiotic mechanisms of action against *C. albicans* are not fully clear [27] but our present results suggest that some of the inhibiting effects are pH-driven. Similar observations have been made before [9,28]. It is thought that colonization of *C. glabrata* and *C. albicans* depends on their ability to cope with the presence of lactic and acetic acids produced by commensal microbiota [35]. Lactobacilli produce different weak organic acids (WOAs), mainly lactic and acetic acids, but the capability and rate of acid production are strain-specific [36]. In our study, *L. rhamnosus* DSM 32992 and *L. rhamnosus* DSM 32991 generally showed the lowest pH values indicating the highest acid production of the selected lactobacilli. The findings correspond well with the results from the growth inhibition test, in which these two strains exhibited the best inhibitory effect. This indicates that acid production plays an important role in the inhibition of *Candida* growth in agar overlay interference assay and indicates that the acids produced by the lactobacilli diffuse into the upper agar layer and interfere with *Candida* growth and survival. Cottier and co-workers [37] investigated the transcriptional stress response of *C. albicans* to WOAs *in vitro* and found the response to be a significant enrichment of genes involved in iron homeostasis, and down-regulation of RNA synthesis and ribosome biogenesis genes. The findings were mostly apparent upon chronic exposure to the acids and suggested that exposure of *C. albicans* to WOAs over time might shift the cells into a ‘starvation-like’ metabolic state with low transcription, translation and growth of the cell [37]. In a study by Köhler et al. [14], *C. albicans* growth was suppressed at low pH by the supernatant of the lactobacilli; however, pH neutralization of the culture filtrate completely abrogated the inhibitory effects of the supernatants. It is likely that low pH inhibits the transition between the blastospore form and the more invasive hyphae form of *C. albicans* and increases the intracellular concentration of protons in the yeasts. This may lead to an increased activity of an energy-consuming plasma membrane H+-ATPase that exhausts the available energy for growth and metabolism, leading to growth inhibition and, finally, cell death of the yeast [38]. Our experiments revealed that the tested *C. krusei* and *C. tropicalis* strains were able to resist and neutralize the acids produced by the lactobacilli. This may be understood in view of an *in vitro* study by Halm et al. [39] who found that lactic acid only induced a short-term (few seconds) pH change intracellularly in *C. krusei*. Fungal resistance to organic acids could be caused by extracellular production of ammonia [40,41], less permeable plasma membranes to lactic acid, higher buffer capacity inside the fungal cells, or higher H+-ATPase capacity [38].

Results from *in vitro* inference studies with single bacteria or multi-species biofilms must be considered with great caution taken the complex environment of the oral cavity into account. The agar overlay interference assay allows screening of multiple strains at a time on a single plate, with different *Lactobacillus* strains in the bottom agar laying at different cell concentrations. The assay is a relatively simple, yet a robust and well-proven method for screening purposes of probiotic bacteria against oral pathogens [9,13,25]. In our experiments, we modified the growth inhibition scoring system to allow for a more sensitive screening. Based on our previous experience [9], we separated the original score 1 (‘slight inhibition’) into score 1 ‘almost total growth inhibition (colonies slightly visible)’ and score 2 ‘slight growth inhibition (colonies are clearly visible but smaller than at the control plate)’. The advantage of this modified system was that we were able to distinguish the growth inhibition capacity of the 14 probiotic candidates but the downside was that the results were harder to compare with the results found in comparable studies using the same inhibition assay [9,13,42].

There is evidence to support the hypothesis that the efficacy of probiotics is both strain-specific and disease-specific [43]. Therefore, our present the finding of two probiotic *L. rhamnosus* candidates (strain-specificity) with excellent antifungal capabilities (disease-specificity) isolated from the oral cavity, the environment where they are intended to exhibit their probiotic action (target site-specificity), was promising. Future studies on the characteristics of the four candidates, or combinations thereof, seems motivated to further unveil the probiotic and physiological properties including the risk of transmission of antibiotic resistance genes. In addition to clinically demonstrated health benefits and safety for human use, the persistence of cell viability and probiotic activities throughout the processing, handling and storage needs to be investigated [31].

## Conclusion

All 14 *Lactobacillus* strains employed in this *in vitro* screening demonstrated antifungal activity and inhibited the growth of six *Candida* spp. to varying degrees. The effect seemed pH-driven and two *L. rhamnosus* strains isolated from the oral cavity showed the strongest growth inhibition and acid production ability among the selected strains. The findings are promising for future clinical employment of these strains in the prevention of oral *C. albicans* and non-*C. albicans* infections; however, further investigations and characterization of the strains are needed to elaborate on their probiotic properties.

## Supplementary Material

Supplemental Material

## References

[1] Rindum JL, Stenderup A, Holmstrup P. Identification of *Candida albicans* types related to healthy and pathological oral mucosa. J Oral Pathol Med. 1994;23:406–8. 7823301 10.1111/j.1600-0714.1994.tb00086.x

[2] Kragelund C. Exploiting new knowledge of *Candida* infection for future antifungal combat. Oral Dis. 2017;23:543–547. 27442140 10.1111/odi.12546

[3] Oever JT, Netea MG. The bacteriome-mycobiome interaction and antifungal host defense. Eur J Immunol. 2014;44:3182–3191. 25256886 10.1002/eji.201344405

[4] Sardi JC, Scorzoni L, Bernardi T, et al. *Candida* species: current epidemiology, pathogenicity, biofilm formation, natural antifungal products and new therapeutic options. J Med Microbiol. 2013;62:10–24. 23180477 10.1099/jmm.0.045054-0

[5] Pfaller MA, Andes DR, Diekema DJ, et al. Epidemiology and outcomes of invasive candidiasis due to non-*albicans* species of *Candida* in 2,496 patients: data from the Prospective Antifungal Therapy (PATH) registry 2004-2008. PLoS One. 2014;9(7):e101510. . 24991967 10.1371/journal.pone.0101510 PMC4081561

[6] U.S. Department of Health and Human Services, Centers for Disease Control and Prevention. Antibiotic resistance threats in the USA 2019. [cited 2020 414]. Available from: https://www.cdc.gov/drugresistance/pdf/threats-report/2019-ar-threats-report-508.pdf

[7] World Health Organization. Antimicrobial resistance global report on surveillance. 2014 [cited 2020 414]. Available from: https://apps.who.int/iris/bitstream/handle/10665/112642/9789241564748_eng.pdf;jsessionid=3DFA76135096412FF7041954E144562E?sequence=1

[8] Kraft-Bodi E, Jørgensen MR, Keller MK, et al. Effect of probiotic bacteria on oral *Candida* in frail elderly. J Dent Res. 2015;94(9 Suppl):181–186. . 10.1177/0022034515595950 26202995

[9] Jørgensen MR, Kragelund C, Jensen PØ, et al. Probiotic *Lactobacillus reuteri* has antifungal effects on oral *Candida* species *in vitro*. J Oral Microbiol. 2017;9:1274582. 28326154 10.1080/20002297.2016.1274582 PMC5328390

[10] Hu L, Mao Q, Zhou P, et al. Effects of *Streptococcus salivarius* K12 with nystatin on oral candidiasis-RCT. Oral Dis. 2019;25:1573–1580. 31177581 10.1111/odi.13142

[11] Sanders ME. Probiotics: definition, sources, selection, and uses. Clin Infect Dis. 2008;46(Suppl 2):S58–61. discussion S144-51. . 18181724 10.1086/523341

[12] Matsubara VH, Bandara HM, Mayer MP, et al. Probiotics as antifungals in mucosal candidiasis. Clin Infect Dis. 2016;62:1143–1153. 26826375 10.1093/cid/ciw038

[13] Hasslöf P, Hedberg M, Twetman S, et al. Growth inhibition of oral mutans streptococci and candida by commercial probiotic lactobacilli - an *in vitro* study. BMC Oral Health. 2010;10:18. 20598145 10.1186/1472-6831-10-18 PMC2908555

[14] Köhler GA, Assefa S, Reid G. Probiotic interference of *Lactobacillus rhamnosus* GR-1 and *Lactobacillus reuteri* RC-14 with the opportunistic fungal pathogen *Candida albicans*. Infect Dis Obstet Gynecol. 2012. DOI:10.1155/2012/636474 PMC3395238 22811591

[15] Ujaoney S, Chandra J, Faddoul F, et al. *In vitro* effect of over-the-counter probiotics on the ability of *Candida albicans* to form biofilm on denture strips. J Dent Hyg. 2014;88:183–189. 24935148

[16] Murzyn A, Krasowska A, Stefanowicz P, et al. Capric acid secreted by *S. boulardii* inhibits *C. albicans* filamentous growth, adhesion and biofilm formation. PLoS One. 2010;5:e12050. 20706577 10.1371/journal.pone.0012050 PMC2919387

[17] Tan Y, Leonhard M, Moser D, et al. Inhibitory effect of probiotic lactobacilli supernatants on single and mixed non-*albicans Candida* species biofilm. Arch Oral Biol. 2018;85:40–45. 29031236 10.1016/j.archoralbio.2017.10.002

[18] Aarti C, Khusro A, Varghese R, et al. *In vitro* investigation on probiotic, anti-*Candida*, and antibiofilm properties of *Lactobacillus pentosus* strain LAP1. Arch Oral Biol. 2018;89:99–106. 29499562 10.1016/j.archoralbio.2018.02.014

[19] Hatakka K, Ahola AJ, Yli-Knuuttila H, et al. Probiotics reduce the prevalence of oral *Candida* in the elderly–a randomized controlled trial. J Dent Res. 2007;86:125–130. 17251510 10.1177/154405910708600204

[20] Ishikawa KH, Mayer MP, Miyazima TY, et al. A multispecies probiotic reduces oral *Candida* colonization in denture wearers. J Prosthodont. 2015;24:194–199. 25143068 10.1111/jopr.12198

[21] Miyazima TY, Ishikawa KH, Mayer M, et al. Cheese supplemented with probiotics reduced the *Candida* levels in denture wearers-RCT. Oral Dis. 2017;23:919–925. 28346730 10.1111/odi.12669

[22] Lee X, Vergara C, Lozano CP. Severity of *Candida*-associated denture stomatitis is improved in institutionalized elders who consume *Lactobacillus rhamnosus* SP1. Aust Dent J. 2019;64:229–236. 30963591 10.1111/adj.12692

[23] Li D, Li Q, Liu C, et al. Efficacy and safety of probiotics in the treatment of *Candida*-associated stomatitis. Mycoses. 2014;57:141–146. 23952962 10.1111/myc.12116

[24] Hu L, Zhou M, Young A, et al. *In vivo* effectiveness and safety of probiotics on prophylaxis and treatment of oral candidiasis: a systematic review and meta-analysis. BMC Oral Health. 2019;19:140. 31291932 10.1186/s12903-019-0841-2 PMC6621984

[25] Simark-Mattsson C, Emilson CG, Hakansson EG, et al. *Lactobacillus*-mediated interference of mutans streptococci in caries-free vs. caries-active subjects. Eur J Oral Sci. 2007;115:308–314. 17697171 10.1111/j.1600-0722.2007.00458.x

[26] Rossoni RD, de Barros PP, de Alvarenga JA, et al. Antifungal activity of clinical *Lactobacillus* strains against *Candida albicans* biofilms: identification of potential probiotic candidates to prevent oral candidiasis. Biofouling. 2018;34:212–225. 29380647 10.1080/08927014.2018.1425402

[27] Ribeiro FC, Rossoni RD, de Barros PP, et al. Action mechanisms on *Candida* spp. and candidiasis prevention: an update. J Appl Microbiol. 2019. DOI:10.1111/jam.14511. 31705713

[28] Jiang Q, Stamatova I, Kari K, et al. Inhibitory activity *in vitro* of probiotic lactobacilli against oral *Candida* under different fermentation conditions. Benef Microbes. 2015;6:361–368. 25380800 10.3920/BM2014.0054

[29] Salari S, Almani PGN. Antifungal effects of *Lactobacillus acidophilus* and *Lactobacillus plantarum* against different oral *Candida* species isolated from HIV/AIDS patients: an *in vitro* study. J Oral Microbiol. 2020;12(1):1769386. 32922676 10.1080/20002297.2020.1769386 PMC7448839

[30] Allonsius CN, van den Broek MFL, De Boeck I, et al. Interplay between *Lactobacillus rhamnosus* GG and *Candida* and the involvement of exopolysaccharides. Microb Biotechnol. 2017;10:1753–1763. 28772020 10.1111/1751-7915.12799 PMC5658588

[31] Capurso L. Thirty years of *Lactobacillus rhamnosus* GG: A review. J Clin Gastroenterol. 2019;53(Suppl 1):S1–S41. 30741841 10.1097/MCG.0000000000001170

[32] Pankhurst CL. Candidiasis (oropharyngeal). BMJ Clin Evid. 2012;2012:1304. PMC3821534 24209593

[33] Ahola AJ, Yli-Knuuttila H, Suomalainen T, et al. Short-term consumption of probiotic-containing cheese and its effect on dental caries risk factors. Arch Oral Biol. 2002;47:799–804. 12446187 10.1016/s0003-9969(02)00112-7

[34] Mailänder-Sánchez D, Braunsdorf C, Grumaz C, et al. Antifungal defense of probiotic *Lactobacillus rhamnosus* GG is mediated by blocking adhesion and nutrient depletion. PLoS One. 2017;12:e0184438. 29023454 10.1371/journal.pone.0184438 PMC5638248

[35] Lourenco A, Pedro NA, Salazar SB, et al. Effect of acetic acid and lactic acid at low pH in growth and azole resistance of *Candida albicans* and *Candida glabrata*. Front Microbiol. 2018;9:3265. 30671051 10.3389/fmicb.2018.03265 PMC6331520

[36] Zangl I, Pap I-J, Aspöck C, et al. The role of *Lactobacillus* species in the control of *Candida* via biotrophic interactions. Microb Cell. 2020;7:1–14. 10.15698/mic2020.01.702 PMC6946018 31921929

[37] Cottier F, Tan AS, Chen J, et al. The transcriptional stress response of *Candida albicans* to weak organic acids. G3 (Bethesda). 2015;5:497–505. 25636313 10.1534/g3.114.015941 PMC4390566

[38] Holyoak CD, Stratfor M, McMullin Z, et al. Activity of the plasma membrane H^+^-ATPase and optimal glycolytic flux are required for rapid adaptation and growth of *Saccharomyces cerevisiae* in the presence of the weak-acid preservative sorbic acid. Appl Environ Microbiol. 1996;62:3158–3164. 8795204 10.1128/aem.62.9.3158-3164.1996 PMC168110

[39] Halm M, Hornbaek T, Arneborg N, et al. Lactic acid tolerance determined by measurement of intracellular pH of single cells of *Candida krusei* and *Saccharomyces cerevisiae* isolated from fermented maize dough. Int J Food Microbiol. 2004;94:97–103. 15172490 10.1016/j.ijfoodmicro.2003.12.019

[40] Zikanova B, Kuthan M, Ricicova M, et al. Amino acids control ammonia pulses in yeast colonies. Biochem Biophys Res Commun. 2002;294:962–967. 12074570 10.1016/S0006-291X(02)00589-2

[41] Vylkova S, Carman AJ, Danhof HA, et al. The fungal pathogen *Candida albicans* autoinduces hyphal morphogenesis by raising extracellular pH. MBio. 2011;2:e00055–11. 21586647 10.1128/mBio.00055-11 PMC3101780

[42] Keller MK, Hasslof P, Stecksen-Blicks C, et al. Co-aggregation and growth inhibition of probiotic lactobacilli and clinical isolates of mutans streptococci: an *in vitro* study. Acta Odontol Scand. 2011;69:263–268. 21306197 10.3109/00016357.2011.554863

[43] McFarland LV, Evans CT, Goldstein EJC. Strain-specificity and disease-specificity of probiotic efficacy: A systematic review and meta-analysis. Front Med. 2018;5:124. 10.3389/fmed.2018.00124 PMC5949321 29868585

