# Supplementary material for: Lactobacillus rhamnosus strains of oral and vaginal origin show strong antifungal activity in vitro
Source: J Oral Microbiol. 2020 Oct 18;12(1):1832832. doi: 10.1080/20002297.2020.1832832 (PMC7594750; doi:10.1080/20002297.2020.1832832)
Supplement: Supplemental Material [file ZJOM_A_1832832_SM5287.docx]

**Supplementary on-line files**

**Table S1.** Growth inhibition (median scores) of six clinical isolates and six reference *Candida* spp. by 14 different *Lactobacillus* spp. at different cell concentrations (colony forming units (CFU)/mL)

**Figure S1**. Frequency in percent (%) of growth inhibition score 0-3 for each dose (10^5^-10^9^ CFU/mL) based on agar overlay interference tests with all 14 *Lactobacillus* spp.

**Figure S2**. Micro-sensor measurement of pH with the four best performing *Lactobacillus* strains in the bottom agar layer without *Candida* spp. inoculated at the top agar layer. Zero on the vertical axis represents the first measurement from the sensor after touching the surface of the agar.

**Table S1.**

|  | | *Candida* species | | | | | | | | | | |  |
| --- | --- | --- | --- | --- | --- | --- | --- | --- | --- | --- | --- | --- | --- |
| *Lactobacillus* species | **CFU/mL** | *C. albicans*  CCUG 46390 | *C. albicans*  clinical strain | *C. dubliniensis* CCUG 48722 | *C. dubliniensis* clinical strain | *C. glabrata*  CCUG 63819 | *C. glabrata*  clinical strain | *C. krusei*  CCUG 56126 | *C. krusei*  clinical strain | *C. parapsilosis* CCUG 56136 | *C. parapsilosis* clinical strain | *C. tropicalis* CCUG 47037 | *C. tropicalis* clinical strain |
| *Lactobacillus crispatus* 23B33 | 10^9^  10^7^  10^5^ | 1  1  2 | 1  2  2 | 2  2  1 | 1  2  2 | 2  2  2 | 1  2  2 | 2  2  **3** | **3**  **3**  **3** | 1  1  2 | 1  1  2 | 2  2  2 | 1  1  2 |
| *Lactobacillus crispatus* NEU458 | 10^9^  10^7^  10^5^ | 1  2  1 | 1  1  2 | 1  1  1 | **0**  1  1 | 2  1  2 | 2  1  1 | 2  2  2 | **3**  2  2 | 1  1  **0** | **0**  **0**  **0** | **3**  **3**  **0** | 1  1  1 |
| *Lactobacillus fermentum* S1P1 | 10^9^  10^7^  10^5^ | 1  1  1 | 1  1  1 | 1  1  1 | 1  1  1 | 2  2  1 | 2  2  1 | 2  2  2 | 2  **3**  2 | 1  1  **0** | **0**  **0**  1 | **3**  **3**  **3** | 1  1  1 |
| *Lactobacillus fermentum* S1P2 | 10^9^  10^7^  10^5^ | 1  1  1 | 1  1  1 | 1  1  1 | 1  1  1 | 1  1  1 | 1  1  1 | 2  2  2 | **3**  2  2 | 1  1  1 | 1  1  **0** | 2  2  **3** | 1  1  1 |
| *Lactobacillus jensenii* 12B1 | 10^9^  10^7^  10^5^ | 2  2  2 | 2  2  2 | 1  1  1 | 1  1  1 | 2  2  2 | 2  2  2 | 2  2  2 | 2  2  **3** | 1  1  1 | 1  1  1 | **3**  **3**  2 | 1  1  2 |
| *Lactobacillus jensenii* 22B42 | 10^9^  10^7^  10^5^ | 1  1  1 | 1  1  1 | 1  1  1 | 1  1  1 | 1  1  1 | 1  1  1 | 2  2  1 | 2  **3**  2 | 1  1  **0** | 1  1  **0** | 2  1  **3** | 1  1  1 |
| *Lactobacillus rhamnosus* PB01 | 10^9^  10^7^  10^5^ | 1  2  2 | 1  1  2 | 1  1  1 | 1  1  1 | 1  1  2 | 1  1  2 | 2  2  2 | 2  2  2 | 1  1  1 | **0**  **0**  1 | **0**  1  1 | 1  1  2 |
| *Lactobacillus rhamnosus* NEU427 | 10^9^  10^7^  10^5^ | 1  2  2 | 1  1  1 | 1  1  2 | 1  1  1 | 2  2  2 | 2  2  2 | 2  2  2 | **3**  **3**  **3** | 1  **0**  1 | **0**  **0**  1 | 2  2  **3** | 1  1  1 |
| *Lactobacillus rhamnosus* ERB18 | 10^9^  10^7^  10^5^ | 1  1  1 | 1  1  1 | 1  1  1 | 1  1  1 | 1  1  2 | 1  1  2 | 2  2  2 | 2  2  2 | 1  **0**  1 | 1  **0**  1 | 1  1  2 | 1  1  1 |
| *Lactobacillus rhamnosus* ERB 36 | 10^9^  10^7^  10^5^ | 1  1  1 | 1  1  1 | 1  1  1 | 1  1  1 | 1  1  2 | 1  1  2 | 1  1  1 | 2  2  2 | 1  1  1 | 1  1  1 | 1  1  2 | 1  1  1 |
| *Lactobacillus gasseri* EB01 | 10^9^  10^7^  10^5^ | 2  1  2 | 2  1  2 | 1  1  2 | 1  1  1 | 1  1  2 | 1  1  1 | 2  2  2 | 2  2  2 | 1  1  1 | 1  1  1 | 1  1  2 | 2  1  2 |
| *Lactobacillus curvatus* EB10 DSM 32307 | 10^9^  10^7^  10^5^ | 2  1  2 | 2  1  2 | 2  1  2 | 2  1  1 | 2  1  2 | 1  1  2 | 2  2  2 | 2  2  2 | 1  1  2 | 1  1  2 | 2  2  2 | 2  1  2 |
| *Lactobacillus acidophilus* EB03 | 10^9^  10^7^  10^5^ | 1  2  2 | 2  2  2 | 1  2  2 | 1  **3**  **3** | 2  2  2 | 2  2  2 | 2  2  2 | **3**  2  2 | 1  1  2 | 1  1  2 | 2  2  2 | 1  2  2 |
| *Lactobacillus paracasei* S1-P3 | 10^9^  10^7^  10^5^ | 1  1  2 | 1  1  2 | 1  1  1 | 1  1  1 | 2  2  2 | 2  2  2 | 2  2  2 | 2  2  2 | 1  1  1 | 1  1  1 | 1  1  2 | 1  1  2 |

The assays were carried out in duplicates and repeated three times on different occasions. The median inhibition scores are: 0=complete inhibition (no visible colonies); 1=almost total inhibition (colonies are slightly visible); 2= slight inhibition (colonies are clearly visible but smaller than at the control plate); 3= no growth inhibition (colonies equal to those at the control plate)

**Figure S1.**

**Figure S2.**
